# A Study of Radiation-Induced Cerebral Vascular Injury in Nasopharyngeal Carcinoma Patients with Radiation-Induced Temporal Lobe Necrosis

**DOI:** 10.1371/journal.pone.0042890

**Published:** 2012-08-13

**Authors:** Jianhong Ye, Xiaoming Rong, Yanqun Xiang, Yigang Xing, Yamei Tang

**Affiliations:** 1 Department of Neurology, Sun Yat-sen Memorial Hospital, Sun Yat-sen University, Guangzhou, Guangdong Province, China; 2 Key Laboratory of Malignant Tumor Gene Regulation and Target Therapy of Guangdong Higher Education Institutes, Sun Yat-sen University, Guangzhou, Guangdong Province, China; 3 Department of Nasopharyngeal Carcinoma, Cancer Center of Sun Yat-sen University, Guangzhou, Guangdong Province, China; NIH, United States of America

## Abstract

**Purpose:**

To investigate radiation-induced carotid and cerebral vascular injury and its relationship with radiation-induced temporal lobe necrosis in nasopharyngeal carcinoma (NPC) patients.

**Methods and Materials:**

Fifty eight NPC patients with radiation-induced temporal lobe necrosis (TLN) were recruited in the study. Duplex ultrasonography was used to scan bilateral carotid arterials to evaluate the intima-media thickness (IMT) and occurrence of plaque formation. Flow velocities of bilateral middle cerebral arteries (MCAs), internal carotid arteries (ICAs) and basal artery (BA) were estimated through Transcranial Color Doppler (TCD). The results were compared with data from 33 patients who were free from radiation-induced temporal lobe necrosis after radiotherapy and 29 healthy individuals.

**Results:**

Significant differences in IMT, occurrence of plaques of ICAs and flow velocities of both MCAs and ICAs were found between patients after radiotherapy and healthy individuals (p<0.05). IMT had positive correlation with post radiation interval (p = 0.049). Compared with results from patients without radiation-induced TLN, the mean IMT was significantly thicker in patients with TLN (p<0.001). Plaques were more common in patients with TLN than patients without TLN (p = 0.038). In addition, flow velocities of MCAs and ICAs in patients with TLN were much faster (p<0.001, p<0.001). Among patients with unilateral TLN, flow velocity of MCAs was significantly different between ipsilateral and contralateral sides to the lesion (p = 0.001).

**Conclusion:**

Thickening of IMT, occurrence of plaque formation and hemodynamic abnormality are more common in patients after radiotherapy, especially in those with TLN, compared with healthy individuals.

## Introduction

Radiotherapy (RT) plays a critical role in the curative treatment of NPC patients. However, the complications of RT, such as radiation-induced brain injury [1] and radiation-induced vascular injury [2], are not negligible and represent a significant source of morbidity. Previous studies had found that patients who underwent irradiation of the head and neck had a higher risk of developing significant carotid stenosis [2], [3], [4] and eventually suffering from transient ischemic attack or stroke. As we know, significant stenosis poses a threat of embolic or ischemic stroke [5], [6]. With the mechanisms underlying radiation-induced brain injury being revealed gradually, injury to the vessels has been given priority in the study of its relationship with brain injury. For patients after RT, are there any differences in the degree of injury of cerebral and carotid arteries between patients with radiation-induced brain injury and those without brain injury? Considering the fact that temporal lobe is the most vulnerable area after RT in NPC patients and often develops necrosis, in this study, we assess the differences in carotid wall thickness (intima-media thickness), incidence of plaque formation and hemodynamics of cerebral and carotid arteries between NPC patients with and without temporal lobe necrosis, and aim to reveal the relationship between vascular injury and temporal lobe necrosis.

## Methods

This project was approved by an authorized human research review board in our institute (Ethics Committee of The Sun Yat-sen University). Patients included in this study were inpatients and outpatients of the Sun Yat-sen Memorial Hospital of Sun Yat-sen University. Written informed consents were obtained from all involved subjects.

### Patients

From January 2007 to December 2009, NPC patients who were admitted in our hospital were taken into consideration. And those fulfilling the following eligibility criteria were recruited in the study as Group 1: (1) a history of NPC with radiotherapy; (2) no evidence of symptomatic recurrent tumor, tumor with brain metastasis, secondary intracranial tumor, brain abscess, cerebral infarction, demyelinating disease, encephalitis or other central nervous system diseases; (3) unilateral or bilateral temporal lobe necrosis detected by MRI scan. Group 1 consisted of 58 patients (42 males, 16 females, age 38.17±5.06 years). Thirty three NPC patients (24 males, 9 females, age 37.70±4.16 years) without temporal lobe necrosis after radiotherapy were recruited as Group 2. Patients who showed any one of the following were excluded from the study: (1) risk factors for vascular diseases, including hypercholesterolemia, hyperglycemia, hypertension, diabetes and history of smoking and alcoholism; (2) diseases that may lead to vasculitis, including tuberculosis, syphilis and arteritis; (3) history of cardiovascular diseases.

Twenty nine healthy individuals (21 males, 8 females, age 38.10±5.35years) who took body check in our hospital in the same period were chosen as control group.

### Patient Characteristics

In patients of Group 1 and Group 2, the age at completion of radiotherapy ranged from 27 to 45 years, mean 38.29±4.66 years. The mean post radiation interval was 4.53±1.69 years (range from 2 to 12 years). While in control group, the mean age was 38.10±5.35 years. The demographic and clinical characteristics among three groups were similar (p>0.05, Table 1).

**Table 1:** Demographic and clinical characteristics of the three groups.

Characteristic	Group 1	Group 2	Control group
Gender(n)			
male	42(72.4%)	24(72.7%)	21(72.4%)
female	16(27.6%)	9(27.3%)	8(27.6%)
P value	>0.05		
Age (year)	38.62±4.93	37.70±4.16	38.10±5.35
P value	>0.05		
Post-radiation interval (year)	4.58±1.89	4.45±1.30	–
P value	>0.05		
Chemotherapy(n)	18(31.0%)	11(33.3%)	–
P value	>0.05		

The common symptoms in patients with temporal lobe necrosis were headache (25, 43.1%), dizziness (19, 32.8%), syncope (17, 29.3%), hemiplegia (16, 27.6%), impaired memory (17, 29.3%).

### Radiation Technique

All patients underwent conventional irradiation with Low-Melting-Point lead block fitful fields. The two-lateral facial-cervical and inferior neck field followed by the facial-neck sub-field were applied. The target areas included the primary tumor plus 1-cm margin (0.5 cm margin posteriorly) and the regional lymphatics. Potential sites of microscopic extension were also covered as follows, parapharyngeal fat spaces, retropharyngeal nodes, posterior third of the nasal cavity, pterygoidfossae, clivus, petrous tips, base of sphenoid sinus, cavernous sinus. The accumulated radiation doses were 68 to 76 Gy (median, 70.2 Gy), with 2 Gy per fraction applied to the primary tumor, 60 to 64 Gy applied to the involved areas of the neck, and 50 Gy applied to the uninvolved areas. The estimated maximal dose to the adjacent brain was 70 Gy–73 Gy. All patients were treated with one fraction daily for 5 days per week. Chemotherapy was provided in 18 patients of Group 1 and 11 patients of Group 2 respectively.

### Ultrasound Technique

All scans were obtained with an LOGIC700 ultrasound scanner (GE Company) by an experienced sonographer who was blinded about the group. Scanned area included proximal portion of the internal carotid on both sides of the neck. IMT and occurrence of plaque formation of the proximal portion of ICAs were estimated.

Trans-link9900 Transcranial Doppler (TCD) (RIMED Company) was used to obtain the flow velocities, and direction in basal artery (BA), bilateral MCAs and ICAs.

### Statistical Analysis

Mann-Whitney U test was used to compare the clinical characteristics, the IMT of ICAs, and flow velocities of cerebral and carotid arteries among Group 1, Group 2 and control group. X^2^ test was performed to test the occurrence of plaque of ICAs among the three groups. Paired sample t-test was run to compare the vessel pathological changes of ICAs and MCAs between both sides. The relationship among post-radiation interval, age and IMT of ICAs was analyzed by using Pearson’s correlation. All tests were two-tailed and a 5% significance level was used for statistical significance. The SPSS for windows, version 13.0 was used for data processing.

## Results

### Pathological Changes of Carotid Arteries

**Figure 1:**
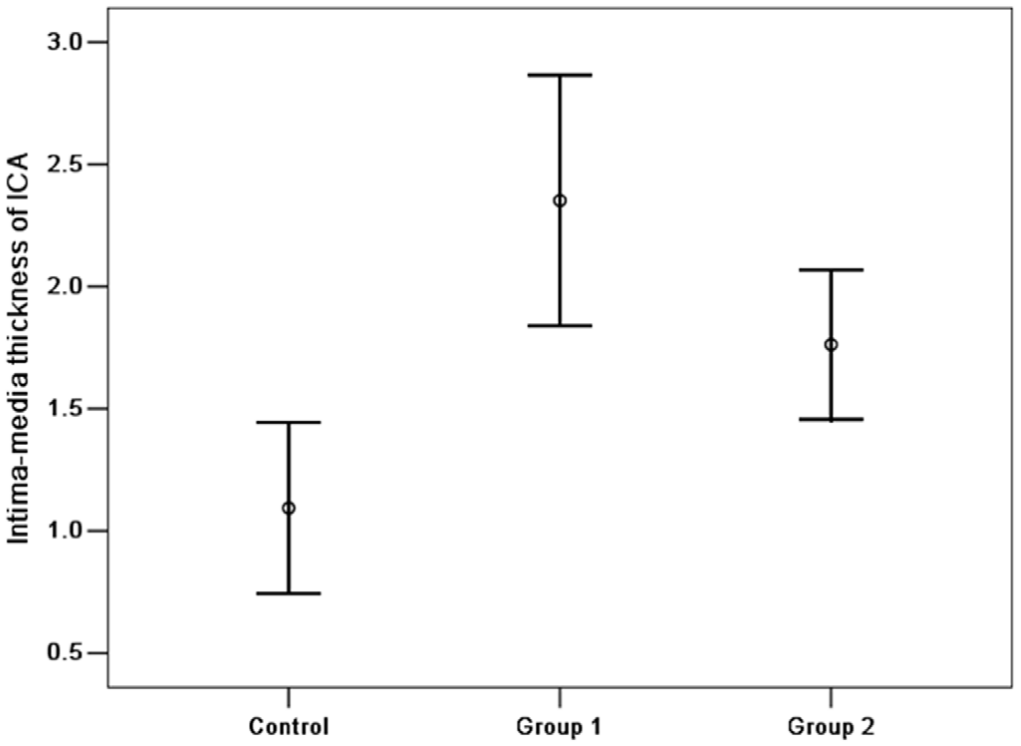
Comparison of IMT of ICAs in the three groups. The results represent with mean±standard deviation.

As presented in Figure 1, in the 58 patients with temporal lobe necrosis, the mean IMT of ICAs was 2.36±0.51 mm, which was significantly thicker than that of the 33 patients without temporal lobe necrosis after radiotherapy (1.70±0.45 mm, p<0.001). Whereas in control group, the mean thickness was 1.10±0.35 mm. The differences between the case groups (Group 1 and Group 2) and control group in the IMT of ICAs were both statistically significant (p<0.001, p<0.001).

IMT was significantly and positively correlated with post radiotherapy interval (Pearson’s correlation coefficient r = 0.368, p = 0.049). There was no correlation between age and IMT (r = 0.331, p = 0.08).

**Figure 2:**
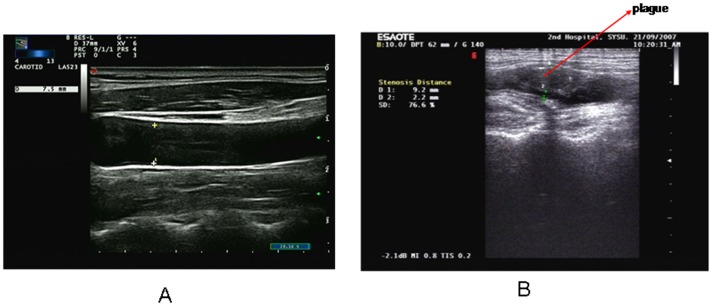
A: Scan from a 40-year-old healthy individual. The diameter of the vessel is 7.5 mm, and the IMT is 0.64 mm. B: Scan from a 38-year old nasopharyngeal carcinoma patient with temporal lobe necrosis after radiotherapy shows narrowed vessel lumen (d = 2.2 mm, IMT = 7 mm) and the atherosclerotic plaque. It shows a more than 76% reduction in luminal diameter. Plaque is displayed with arrowhead.

In the 91 patients after RT, the overall prevalence of plaque formation in ICAs was 42.3%, which was significantly higher than that in control group (42.3% vs. 15.5%, p<0.001). Further more, plaques were more common in patients with temporal lobe necrosis than those without temporal lobe necrosis (44.8% vs. 37.9%, p = 0.038) (Table 2). Figure 2 showed the ultrasound image of ICAs of a healthy individual and a patient with temporal lobe necrosis.

**Table 2:** Prevalence of plaque in ICAs in the three groups.

	No (%)		
Plaque	Group 1	Group 2	Control group
	(n = 58)	(n = 33)	(n = 29)
RICA	28(48.3)	12(36.4)	6(20.7)
LICA	24(41.4)	13(39.4)	3(10.3)
TOTAL	52(44.8)	25(37.9)	9(15.5)

Group 1 = patients with radiation-induced temporal lobe necrosis; Group 2 = Patients without radiation-induced temporal lobe necrosis; Abbreviation: RICA =  right internal carotid artery; LICA = left internal carotid artery.

**Figure 3:**
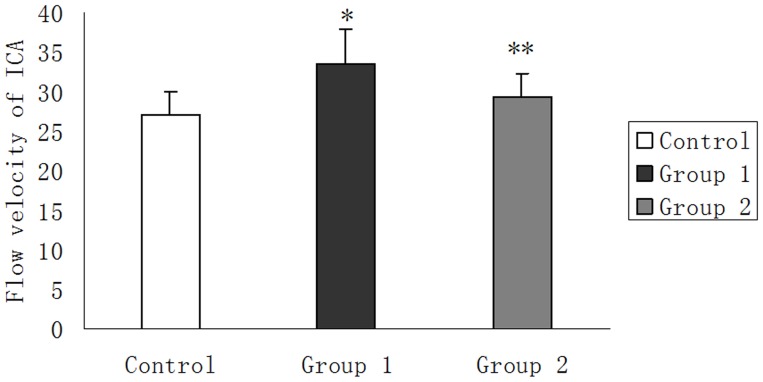
Flow velocities of ICAs among three groups. *Significant difference is found between Group 1 and control group (p<0.001). **Significant difference is found between Group 2 and control group (p<0.001).

Flow velocities of ICAs in Group 1, Group 2 and control group were 33.62±4.23 cm/s, 29.38±2.97 cm/s and 27.17±2.79 cm/s respectively. Flow velocities showed much faster in Group 1 than that in Group 2 (p<0.001). Significant differences were also found between patients after radiotherapy (either Group 1 or Group 2) and healthy individuals (p<0.001, p<0.001) (Figure 3).

### Pathological Changes of Cerebral Arteries

**Figure 4:**
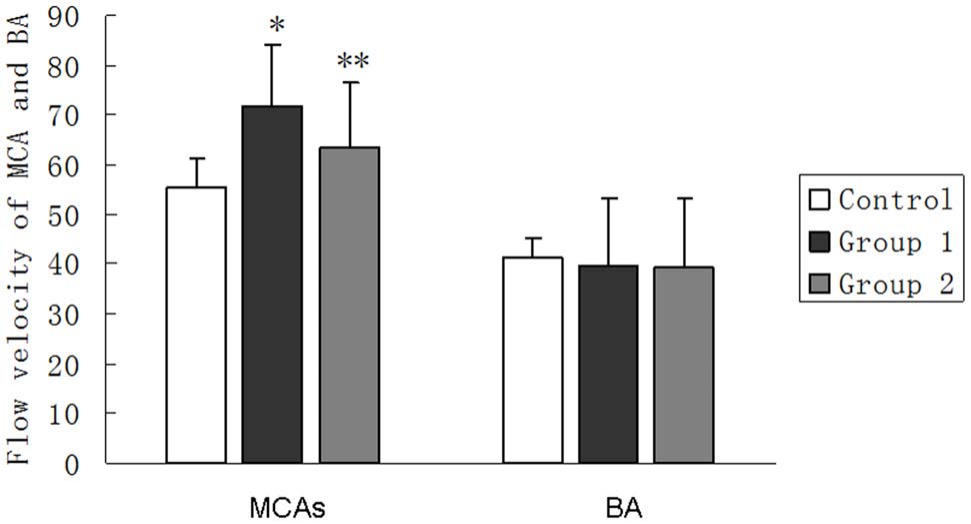
Flow velocities of MCAs and BA among three groups. *The flow velocities of MCAs in Group 1 are much faster than those in control group (p<0.001). ** The flow velocities of MCAs in Group 2 are much faster than those in control group (p<0.001). Also, there is significant difference in MCAs flow velocities between Group 1 and Group 2 (p<0.001).

As shown in Figure 4, the mean flow velocities of vertebrobasilar arteries among Group 1, Group 2 and control group did not show significant difference (39.72±13.58 cm/s, 39.21±13.83 cm/s, 41.21±4.07 cm/s; Group 1 vs. Group 2, p = 0.888; Group1 vs. control group, p = 0.062; Group 2 vs. control group, p = 0.358).

Marked increase in flow velocities of MCAs was observed in Group 1 (71.96±12.08 cm/s), followed by Group 2(63.21±13.19 cm/s). Both of them were significantly higher (p<0.001) than that in control group (55.21±6.02 cm/s) (Figure 4).

### Comparison of Vascular Injury of ICAs and MCAs of Both Sides in Patients with Temporal Lobe Necrosis

Among the 58 patients with temporal lobe necrosis, 28 patients presented unilateral lesions while 30 patients presented bilateral lesions. In the 28 patients with unilateral lesions, the flow velocities of MCAs ipsilateral to the temporal lobe necrosis showed faster than that of contralateral side (non-lesion side) (79.48±10.79 cm/s vs. 68.07±13.67 cm/s, p = 0.001). Yet, the IMT of ICAs of the lesion side did not show significant difference from that of the non-lesion side (2.34±0.57 mm vs. 2.46±0.43 mm,P = 0.355). Also no significant differences were found in prevalence of plaque formation (46.4% vs. 48.3%, p = 0.789) as well as flow velocities of ICAs (32.97±4.72 cm/s vs. 33.79±3.97 cm/s, p = 0.514).

In the 30 patients with bilateral temporal lobe necrosis, no significant difference between bilateral vessels in terms of IMT, prevalence of plaque formation, flow velocities of MCAs as well as ICAs were found (p = 0.872, p = 0.197, p = 0.734, p = 0.627).

## Discussion

This study assessed the IMT, plaque formation and flow velocities of NPC patients after RT. Comparison analysis were made among NPC patients with and without temporal lobe necrosis and healthy individual control. Results showed that there were significant differences in terms of IMT, occurrence of plaque formation and flow velocities between patients after radiotherapy and healthy individual control. Our results support the knowledge that radiotherapy can cause vascular injury. Previous studies [2] showed that the common/internal carotid arteries (CCAs/ICAs) were the most vulnerable arteries after radiation. Radiation dose [1], [7] and post radiation interval [3], [8] are both risk factors for carotid stenosis. A prospective study [9] compared IMT on the same patients before and one year after RT, and found that significant increase of IMT occurred at the first year after RT and continued to increase when completing 24 months of follow-up. In our study, significant differences of flow velocities were found in both MCAs and ICAs, but not BA, among the three groups. The fact that BA is out of the fields of radiation may be the underlying reason of this phenomena. Also, the results demonstrated that patients with RT had an increase in IMT, and the degree of IMT had positive correlation with post radiation interval, which was in line with the previous studies [8], [9], [10], [11], [12]. Irradiation may directly damage the endothelium, lead to the adherence of platelets, and secondary promote the proliferation and migration of smooth muscle cell [13], eventually cause thickening of the arterial wall and even stenosis or occlusion of the arteries. Experimental evidence has been obtained to support the theory that endothelium is the primary target cell population of radiation damage [14]. Radiation-induced stenotic lesions have specific characteristics [15]. The lesions exist in a wide range of carotid artery, and show maximal stenosis near the end of the stenotic area. Increase of IMT and carotid stenosis could both lead to ischemic stroke [4]. Increased of IMT may cause ischemic stroke through change of flow not only in the stenotic vessels but also the circle of Willis [16], which can explain the fact that some patients develop bilateral abnormality of flow. On the other hand, manifestations such as dizziness, syncope and hemiplegia are more common in the population of patients with temporal lobe necrosis in our study, which also suggest vascular injury in these patients, since heart diseases have been excluded.

The interval to diagnosis of temporal lobe necrosis in our study is 4.53±1.69 years. This period represents the interval between completion of radiotherapy and the time when the patient was admitted in our hospital, but not the real latency of temporal lobe necrosis, since some patients are asymptomatic. Therefore it is reasonable that the interval in our study is longer than previous study, which reported a median latency of 33 months [17].

We also found that there were significant differences between patients with temporal lobe necrosis and those without temporal lobe necrosis after RT in IMT, prevalence of plaque formation, as well as flow velocities in MCAs and ICAs. The results indicate that severity of vascular injury is likely to correlate with temporal lobe necrosis. It has been suggested that late radiation effect on normal tissues is related to damage to various critical cell systems, and damage to vascular endothelium plays the primary role in the development of late radiation-induced tissue injury [18]. Yet until now, there is no direct evidence showing the effect of large arterial injury on radiation-related injury. Whether vascular changes are just a marker for late tissue damage or a cause need to be further studied. In our study, a comparison analysis of pathological changes between bilateral vessels in patients with unilateral temporal lobe necrosis showed that no significant differences were found in terms of IMT, plaque formation on ICAs. This indicates that carotid arterial injury is not parallel with temporal lobe injury. Yet, marked increase of flow velocities of MCAs was observed in ipsilateral to the lesion than that in contralateral side. Previous study [19] compared cerebral blood flow between symptomatic group (stroke or TIA) and asymptomatic group and found that significantly lower blood flow in symptomatic patients. This was different from our results in terms of change of blood flow. Considering temporal atrophy, cystic formation, liquefaction and infarction maybe have dissimilarity of manifestations in vascular injury, further studies need to be proceeded.

Our research also revealed that plaques were more common in patients post RT, especially in those with temporal lobe necrosis. Decreasing the risk of complications such as stroke and TIA by using antiplatelet drugs, endarterectomy [20], carotid angioplasty and stenting (CAS) [21] has been proposed as alternative treatment for carotid artery stenosis. Further identification of the role of large vascular injury in the development of temporal lobe necrosis will allow for potential therapy in the future.

Except for radiation strategy (including total dose, radiation fields and so on), systemic diseases and unhealthy living habits such as hypertension, diabetes, and smoking have close relationship with vascular injury. The relationship between those vessel risk factors and radiation, and how they affect the development of temporal lobe necrosis need to be further studied. In addition, prospective study with drug interference may help to find out the role of large vascular injury on temporal lobe necrosis.
